# A Bacterial-Sourced Protein Diet Induces Beneficial Shifts in the Gut Microbiome of the Zebrafish, *Danio rerio*

**DOI:** 10.1016/j.cdnut.2024.102077

**Published:** 2024-01-10

**Authors:** George BH Green, Michael B Williams, Jeri L. Brandom, Sophie B Chehade, Christian X Fay, Casey D Morrow, Addison L Lawrence, Asim K Bej, Stephen A Watts

**Affiliations:** 1 Department of Biology, The University of Alabama at Birmingham, Birmingham, AL, United States; 2 Department of Genetics, University of Alabama at Birmingham, Birmingham, AL, United States; 3 Department of Cell, Developmental and Integrative Biology, The University of Alabama at Birmingham, Birmingham, AL, United States; 4 Texas A&M AgriLife Extension Agriculture and Life Sciences, TAMU College Station, TX, United States; 5 J. Frank Barefield, Jr. Department of Criminal Justice, The University of Alabama at Birmingham, Birmingham, AL, United States

**Keywords:** alternative protein sources, animal nutrition, aquatic animals, nutrition, obesity

## Abstract

**Background:**

Bacterial-sourced single-cell proteins (SCPs) offer an alternative protein source for diet formulation for Zebrafish (*Danio rerio)* and other aquaculture models. In addition, the use of a single-cell bacterial protein source derived from multiple species provides a unique insight into the interplay among nutrients in the diet, microbial populations in the diet, and the gut microbiome in *D. rerio*.

**Objective:**

Our objective in this study was to evaluate the impact of dietary replacement of fish protein hydrolysate in a standard reference (SR) with a single-cell bacterial protein source on *D. rerio* gut microbiome.

**Methods:**

We investigated gut microbial compositions of *D*. *rerio* fed an open-formulation standard reference (SR) diet or a bacterial-sourced protein (BP) diet, utilizing microbial taxonomic co-occurrence networks, and predicted functional profiles.

**Results:**

Microbial communities in the SR diet were primarily composed of Firmicutes. In contrast, the BP diet was mainly composed of Proteobacteria. Alpha diversity revealed significant differences in microbial communities between the 2 diets, and between the guts of *D. rerio* fed either of the 2 diets. *D. rerio* fed with the SR diet resulted in abundance of *Aeromonas* and *Vibrio*. In contrast, *D. rerio* fed with a BP diet displayed a large abundance of members from the Rhodobacteraceae family. Taxonomic co-occurrence networks display unique microbial interactions, and key taxons in *D. rerio* gut samples were dependent on diet and gender. Predicted functional profiling of the microbiome across *D. rerio* fed SR or BP diets revealed distinct metabolic pathway differences. Female *D. rerio* fed the BP diet displayed significant upregulation of pathways related to primary and secondary bile acid synthesis. Male *D. rerio* fed the BP diet revealed similar pathway shifts and, additionally, a significant upregulation of the polyketide sugar unit biosynthesis pathway.

**Conclusions:**

The use of a BP dramatically affects the composition and activity of the gut microbiome. Future investigations should further address the interplay among biological systems and diet and may offer insights into potential health benefits in preclinical and translational animal models.

## Introduction

The availability of quality macronutrients, including protein, is important in the formulation of consistent standard reference (SR) diets in the Zebrafish *Danio rerio* and other model organisms. Most aquatic animal diets use a fish meal or a more purified derivative, such as fish protein hydrolysate, as a predominant protein source. A possible alternative source is single-cell protein (SCP), an ingredient whose content is usually derived from bacteria, microalgae, or yeast. Several SCPs have been shown to support growth and reproductive success when used as a complete or partial replacement for fish protein sources in experimental diets [1]. Interestingly, some of these SCP diets show potential in improving animal health [[1], [2], [3]]. One such SCP diet resulted in reduced adiposity, increased lean matter, and lower concentrations of cholesterol transcripts in *D*. *rerio* [4]. Some SCPs may confer health benefits by affecting the organismal gut microbiome; however, such potential impacts on the microbiome remain largely unexplored.

Similar to humans, *D. rerio* harbors a diverse population of microorganisms within their digestive tract [5]. The digestive system of *D. rerio* shares anatomic and physiologic characteristics generally comparable with the mammalian digestive system. The *D. rerio* gut is segmented into 3 parts: the anterior intestinal bulb, the middle intestine, and the posterior intestine. These segments share similar transcriptomic profiles and digestive physiology with mammals [6]. As a consequence, *D. rerio* can be used to study the role of the gut microbiota in the maintenance of host health, metabolism, or manifestation of disease states due to dysbiosis [6,7]. Additionally, *D. rerio* consumes diets with macronutrient profiles comparable with human consumption and, as such, provides a model in which to investigate the effects of dietary ingredients, associated macro- and micronutrients, and bioactive components on microbial composition and metabolic homeostasis.

The gut microbiome colonization cycle in *D. rerio* differs from humans. Humans initially develop their microbiome inside the womb and within the early years of development [8]. *D. rerio* obtains microorganisms present in their environment followed by the adaptation and establishment of the core microbiome in the gut [9,10]. Despite the differences in the colonization process, several gene regulatory pathways of the gut microbiota in *D. rerio* are conserved in humans, particularly nutrient and xenobiotic metabolism, epithelial cell turnover processes, and innate immune responses [11,12]. In general, laboratory-reared *D. rerio* display gut microbiota dominated by Proteobacteria, followed by Fusobacteria, Firmicutes, Actinobacteria, and Bacteroidetes at the higher taxonomic level [9,13], although evidence of much greater diversity of taxa at the lower taxonomic levels has also been reported [9].

In laboratory husbandry, *D. rerio* are normally fed a combination of live and/or formulated commercial diets. These diets, although largely effective in promoting weight gain, lack nutritional balance, standardized ingredients, and consistency in macronutrient sourcing and diet formulation [12,14,15]. Such inconsistencies in dietary ingredients can result in poor dietary intake and can contribute to dysbiosis resulting in an imbalance in the host metabolism, intestinal and extraintestinal disorders, pathogenesis, and progression of disease [[16], [17], [18]]. This metabolic instability could affect experimental reproducibility among different laboratories, or even within the same laboratory [19].

A consistent observation in the use of *D. rerio* in gut microbiome studies across research laboratories is the individual variation among samples in studies [19]. Similar to humans, *D. rerio* display individualized unique microbial compositions [20]. *D. rerio* unique microbial communities can potentially be explained in part by variation in diet formulations [21]. Green et al. [21] revealed that macronutrient-limited diets fed to *D. rerio* resulted in changes in body weight, adiposity, microbial composition (and associated metabolic function), potentially leading to proinflammatory states. Thus, an appropriate composition of the macro- and micronutrients of *D. rerio* diets is essential for maintaining a stable, healthy gut microbiota and host metabolism.

As previously reported by Watts et al. [22], the quality of protein affects growth outcome and metabolic stability/health in *D. rerio*; therefore, quality and the source of protein must also be considered within the context of the hypothesis in the experimental design. Given the high protein content of a bacterial-sourced SCP, the utilization of bacterial-sourced protein as an ingredient should be considered in animal diets. The use of bacterial-sourced protein has been shown to improve commercial shrimp diets [1] and is effective as a replacement of fish protein in *D. rerio* [23]. In our laboratory, Williams et al. [4] analyzed the effect of bacterial-sourced protein on body metrics, reproduction, and bulk RNA sequencing of the liver in *D. rerio*, revealing not only positive growth profiles but also potential health benefits.

These physiologic and health-related observations by Williams et al. [4] prompted further investigations to determine if the observed changes were associated with changes in gut microbiome. We evaluated an SR diet using fish protein hydrolysate (FPH) as an established protein source, and a potential new protein source using SCP in replacement of FPH with respect to gut microbiome in laboratory-reared *D. rerio.* We reveal a comparative outlook of the gut microbial community compositions between *D. rerio* along with taxonomic co-occurrence networks and predicted metabolic profiles. We employed 16S rRNA gene-based microbiome analysis and bioinformatics tools [21] to address similarities and differences of *D. rerio* gut microbiota fed a BP diet compared with the SR diet. The outcome of this study will help provide novel findings as to the effects of a bacterial-sourced protein on the microbiome and may lead to a fundamental understanding of the value of this ingredient in formulated dietary standards for *D. rerio.*

## Methods

### Diet preparation

Two diets were prepared, an SR diet [4], and an experimental diet (BP) where a bacterial protein source was substituted for FPH (Table 1). Each diet was produced from cholesterol, menhaden oil, corn oil, vitamin (custom vitamin mixture, MP Biomedicals) mineral premixes (MP Biomedicals 290284), alginate binders (ingredients and catalog numbers listed in Williams et al. [4], and casein, a supplementary source of essential amino acids, (MP Biomedicals, Cat. no 0296012805). An additional protein source was FPH (the Scoular Company, Cat. no CPSP90), or the bacterial SCP source provided by Meridian Biotech, LLC. Per Meridian Biotech, the bacterial SCP source was derived from aquatic environmental samples, cultured in a bioreactor, and dried to <10% water content (proprietary microbial biologics). Although this protein source is produced as dried biomass, populations of specific species may be active. All ingredients were weighed on an analytical balance (Mettler Toledo New Classic MF Model MS8001S or Model PG503-S Mettler-Toledo, LLC) and mixed using a Kitchen Aid Professional 600 Orbital Mixer (Kitchen Aid). The diets were cold extruded to preserve nutrient content into strands using a commercial food processor (Cuisinart), and strands were air-dried for 24 h on wire trays. A proximate analysis of the 2 diets was performed by Eurofins.

**TABLE 1:** Composition of diets used for feeding trial

Ingredients (g/kg)	SR	BP
Casein – low trace metals	350.00	350.00
Fish protein hydrolysate	200.00	0.00
MRD Pro Batch 2	0.00	317.90
Wheat starch	56.50	56.50
Dextrin type III	16.10	16.10
Alfa cellulose	10.00	10.00
Diatomaceous earth	125.70	0.00
Menhaden fish oil (ARBP) Virginia Prime Gold	26.00	39.00
Safflower oil	45.50	40.30
Alginate	20.00	20.00
Soy lecithin (refined)	40.00	40.00
Vit Pmx (MP Vit Diet Fortification Mixture)^1^	40.00	40.00
Mineral Pmx aka premix (AIN 93G)^2^	30.00	30.00
Canthaxanthin (10%)	23.10	23.10
Potassium phosphate monobasic	11.50	11.50
Glucosamine	2.50	2.50
Betaine	1.50	1.50
Cholesterol	1.20	1.20
Ascorbylpalmitate	0.40	0.40
Total	1000.00	1000.00

1 MP Biomedicals 904654: vitamin A acetate (500,000 IU/g) 1.80000, vitamin D2 (850,000 IU/g) 0.12500, DL-α-tocopherol acetate 22.00000, ascorbic acid 45.00000, inositol 5.00000, choline chloride 75.00000, menadione 2.25000, p-aminobenzoic acid 5.00000, niacin 4.25000, riboflavin 1.00000, pyridoxine hydrochloride 1.00000, thiamine hydrochloride 1.00000, calcium pantothenate 3.00000, biotin 0.02000, folic acid 0.09000, vitamin B12 0.00135, measures are mg/g.

2 AIN 93 mineral mix for Envigo: sucrose, fine ground 209.496, calcium carbonate 357.0, sodium chloride 74.0, potassium phosphate, monobasic 250.0, potassium citrate, monohydrate 28.0, potassium sulfate 46.6, magnesium oxide 24.3, manganese carbonate 0.63, ferric citrate 6.06, zinc carbonate 1.65, cupric carbonate 0.31, potassium iodate 0.01, sodium selenite 0.0103, chromium potassium sulfate, dodecahydrate 0.275, lithium chloride 0.0174, boric acid 0.0815, sodium fluoride 0.0635, nickel carbonate hydroxide, tetrahydrate 0.0318, ammonium meta-vanadate 0.0066 measures are mg/g.

### Experimental housing and husbandry

The experimental procedures used for this vertebrate animal study on *D. rerio* were approved via University of Alabama at Birmingham (UAB) Institutional Animal Care and Use Committee (IACUC) and adhere to standardized *D. rerio* husbandry requirements for housing and euthanasia under the permit IACUC-20656, 10/29/14, S.A. Watts. *D. rerio* embryos (AB strain) were collected randomly from a mass spawning of males and females. The embryos collected were transferred to Petri dishes (*n* = 50 per dish) and were incubated at 28.5°C until 5 d postfertilization (dpf). At the 5 dpf time point, the hatched larvae were polycultured in 6-L static tanks (*n* = 100 larvae per tank) with the rotifer *Branchionus plicatilis* L-type (Reed Mariculture), which were kept at a salinity of 5 ppt and fed a blend of 6 enriched microalgae (RotiGrow Plus, Reed Mariculture). At the 11-dpf time point, all tanks were fixed onto a recirculating aquaculture system (ZS560 Standalone System, Aquaneering) and fed stage-1 Artemia nauplii until 28 dpf. At 28 dpf, fish from all the 6-L tanks were combined, and randomly placed into 2.8L tanks at *n* = 14 fish per tank. Before the feeding trial, initial weights were determined via subsampling *D. rerio* (*n* = 128) and collecting those individual weights (average initial wet weight = 53 mg). Tanks were randomly assigned to 1 of the 2 diet treatments (*n* = 10 tanks per treatment), and the feeding trial started. *D. rerio* was placed on the respective diet treatments for 16 wk. During the initial 2 wk of the trial, *D. rerio* received powdered feeds, which were provided at a ration of 10% of initial body weight per day. The daily rations were weighed for each tank. The rations were adjusted based on weight gain and food conversion ratios every 2 wk. *D. rerio* was fed at 08:00 and 16:00 each day (United States Central Time).

All tanks were maintained at ∼28°C and ∼1500 μS/cm conductivity in a commercial recirculating system (ZS560 Standalone System, Aquaneering), which completed 5.4 L exchanges from each tank per hour. The water was sourced from municipal tap water and was passed through mechanical filtration (1-μm sediment filter), an activated carbon filter, a reverse osmosis filter, and a cation–anion exchange resin. Synthetic sea salts (instant ocean, Marinemix) were added to adjust the conductivity of the system water. To maintain a pH of 7.4, sodium bicarbonate was added. The total ammonia nitrogen, nitrite, and nitrate were measured colorimetrically once weekly. To sustain adequate water quality, a water exchange of ∼10% was performed on the recirculating system daily. The water passes through an activated charcoal filter, and UV sterilization (provide wavelength and if possible, intensity) on each pass through the system before it re-enters tanks to reduce potential persistent compounds from feed or microbial organisms. Tanks are maintained on the same recirculating system throughout the experiment. To reduce confounding effects from environmental noise, light, vibration, or other unidentified sources, tanks were cleaned and returned to a randomly assigned new position on the recirculating system every 2 wk. Experimental *D. rerio* were maintained under a 14-h light/10-h dark cycle with lights turned on at 07:00 local time (United States Central Time).

### Sample preparation for high-throughput sequencing

At the termination of the 16-wk feeding trial, *D. rerio* whole guts (intestine) of 3 males and 3 females from each dietary treatment were used for high-throughput sequencing with sample destinations as follows: female *D. rerio* fed with the SR diet (SR F); male *D. rerio* fed with the SR diet (SR M); female *D. rerio* fed with the BP diet (BP F); and male *D. rerio* fed with the BP diet (BP M). Intestines were dissected, frozen in liquid nitrogen, and then stored at −80°C. Total DNA was isolated from whole gut samples using Quick DNA Fecal/Soil Microbe Miniprep (Cat# D6010, ZYMO Research) per the manufacturer’s instructions. Purified DNA was subjected to quantification and purity assessment via an Epoch microplate spectrophotometer (BioTek Instruments). Additionally, the BP diet and SR diet were sampled (*n* = 3 samples) as above for high-throughput sequencing.

### High-throughput sequencing

The high-throughput amplicon sequencing was performed on an Illumina MiSeq using the 250 bp paired-end kits (Illumina, Inc.) and by targeting the V4 hypervariable region of the bacterial 16S rRNA gene. The resultant sequences were demultiplexed, and FASTQ formatted [24,25] and then deposited on the National Center for Biotechnology Information Sequence Read Archive under BioProject #PRJNA958190 for the *D. rerio* male and female fed with the BP diet and male and female *D. rerio* fed with the SR diet. The subgroups were labeled for this study as BP female (*n* = 3) and BP male (*n* = 3) for *D. rerio* fed with the bacterial protein diet, and for the SR diet, females and males were labeled as SR female (*n* = 3) and SR male (*n* = 3). Additionally, the BP diet was labeled BP diet (*n* = 3), and the SR diet was labeled SR diet (*n* = 3).

### Taxonomic distribution

The taxonomic profiles of the BP diet group and the SR diet group fed *D*. *rerio* were determined via QIIME2 (2023.2) [26]. Initial FASTQ files were imported into QIIME2 (2023.2) [26] via “qiime tools import” using the cassava 1.8 paired-end demultiplexed fastq format (input format CasavaOneEightSingleLanePerSampleDirFmt). The raw data were subjected to quality checking using the “qiime demux summarize” function. This was followed by quality filtering via DADA2 (q2-dada2 denoize-paired) [27]. The denoizing results from DADA2 were summarized via the “qiime feature-table summarize” command. Then the representative sequences were generated (q2- feature-table tabulate-seqs). The DADA2 statistics were generated via the “qiime metadata tabulate.” The mafft program (q2-alignment) aligned the amplicon sequence variants (ASVs) [28] and piped into fasttree2 (q2-phylogeny) to build a phylogeny [29] using the default building method. To generate alfa diversity (Faith’s Phylogenetic Diversity [30]), beta diversity [31], unweighted UniFrac [31], Jaccard distance, Bray–Curtis dissimilarity, principal coordinate analysis (PCoA), Simpson [32], and Shannon [33] metrics the core–metrics–phylogenetic command via “q2-diversity plugin” was used [34]. The samples were rarefied to a minimum of 40041 sequences per sample, subsampled without replacement. Taxonomic IDs were assigned to ASVs via the command q2-feature-classifier [35] plugin utilizing “classify-sklearn” against the silva-138-99-nb-classifier [36]. The taxonomy generated was collapsed into levels [[1], [2], [3], [4], [5], [6], [7]] and piped into table format using “qiime taxa collapse” [26]. The q2-diversity plugin [35] was utilized to generate ANOSIM statistics via “beta-group-significance,” as well as adonis values via the “adonis” command [34].

### Co-occurrence analysis of microbial taxa

Significant co-occurrence patterns between the microbial communities in the *D. rerio* intestine samples were determined via the Co-occurrence Network interface (CoNet v1.1.1) [33,[37], [38], [39]]. The ASV table and appropriate names of taxa were uploaded in Cytoscape (v3.8.0) [38,39] via the CoNet (v1.1.1) plugin. The connection to higher-level taxa was not explored, and a parent–child exclusion was applied to the data. The *D. rerio* taxonomic input with an accumulative sum of 200, and with at least 2/3 of the samples with nonzero values, were kept. To determine significant co-occurrences [37,38,[40], [41], [42], [43], [44], [45], [46]] between taxa, a 10^8^ pseudocount was applied. The union method was selected to combine the 200 lowest (most negative) and 200 highest (most positive) edges via the mean value [41]. For randomization, the multiedge scores were shuffled row-wise at 100 permutations. The node pairs were then merged (Brown method), which were assigned via the *P* values of the multiedges. To establish the *q* value (corrected significance value), a threshold of *P* value of <0.05 was applied to determine the significance [38,39], and unstable edges were filtered out. The finalized network was established in Cytoscape (v3.8.0), using the radial layout via the yFiles plugin (v1.0) [47]. To determine the topologic parameters, NetworkAnalyzer (v2.7) [48] was implemented. Edges were scaled via the *q* value and were colored via their negative (co-exclusion; red) and positive (copresence; green). The nodes were scaled via group abundance size. NetworkAnalyzer (v2.7) determined nodes with a significantly high degree (number of edges), low-betweenness centrality, and closeness centrality, which have been described elsewhere as key taxa [41,[49], [50], [51]]. These features were then plotted as a scatterplot (*y* = closeness centrality; *x* = betweenness centrality) via Microsoft Excel Software. The top 5 features were selected as likely key taxa based on their closeness centrality scores.

#### Predicted functional analysis

Phylogenetic Investigation of Communities by Reconstruction of Unobserved States (PiCRUSt2, v2.5.2) [52] determined the predicted functional profiles/capacity of the gut microbiota across *D. rerio* samples. The command “picrust2_pipeline.py” outputted hidden-state prediction of genomes, metagenome prediction, sequence placement, pathway-level prediction, and Nearest Sequenced Taxon Index values. The descriptions were added to the metagenome predictions via the “add_descriptions.py” command, which describes each functional capacity [52]. The Kyoto Encyclopedia of Genes and Genomes (KEGG) functional profiles were obtained utilizing “custom_map_table” against the KEGG profile descriptions provided in PiCRUSt2. The KEGG output was analyzed via the DeSeq2 package in R [53], which determined differential functional abundances between *D. rerio* fed with the BP diet and *D. rerio* fed with the SR diet.

## Results

### Read quality and sample statistics

The paired-end Illumina MiSeq analysis of the V4 segment of the 16S rRNA gene amplicons generated a raw sequence count and yielded 1,071,083 reads following dada2 quality checking. A total of 2039 observed ASVs were identified after QIIME2 (v2023.2) quality filtering process (Table 2). The observed taxonomic distribution is presented in Supplemental Table 1.

**TABLE 2:** Denoizing statistics after DADA2 in QIIME2 (v2023.2) across all samples

Sample ID	Input	Filtered	Input passed filter (%)	Denoised	Merged	Input merged (%)	Nonchimeric	Input nonchimeric (%)
BP_Diet1	100363	52295	52.11	51457	49633	49.45	48691	48.51
BP_Diet2	107521	51915	48.28	50978	49002	45.57	48403	45.02
BP_Diet3	90317	48554	53.76	47760	45949	50.88	45197	50.04
BP_F1	152600	113889	74.63	113149	108165	70.88	108029	70.79
BP_F2	134739	105079	77.99	103834	94996	70.5	94148	69.87
BP_F3	78734	48034	61.01	47088	43842	55.68	43640	55.43
BP_M1	137795	108030	78.4	106922	97146	70.5	95976	69.65
BP_M2	85159	70202	82.44	69466	67687	79.48	66841	78.49
BP_M3	69380	49823	71.81	49145	47331	68.22	47225	68.07
SR_Diet1	91099	52513	57.64	51099	47598	52.25	46451	50.99
SR_Diet2	89571	46070	51.43	45033	41937	46.82	40600	45.33
SR_Diet3	91643	46289	50.51	45111	41755	45.56	40041	43.69
SR_F1	66582	47522	71.37	46487	43503	65.34	43450	65.26
SR_F2	86904	64606	74.34	63466	59655	68.64	59578	68.56
SR_F3	103603	77501	74.81	76687	72947	70.41	71842	69.34
SR_M1	74874	44652	59.64	43925	41342	55.22	41342	55.22
SR_M2	112802	87678	77.73	86864	82942	73.53	81729	72.45
SR_M3	66263	51087	77.1	50839	49213	74.27	47900	72.29

Paired-end Illumina MiSeq analysis of the V4 segment of the 16S rRNA gene amplicons generated a raw sequence count and yielded 1,071,083 reads following dada2 quality checking. A total of 2039 observed amplicon sequence variants were identified after QIIME2 (v2023.2) quality filtering process.

### Taxonomic distribution across all samples

**FIGURE 1:**
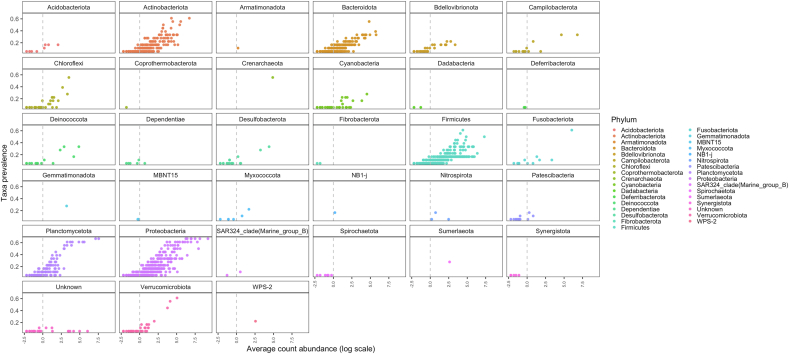
Taxa prevalence was calculated across all samples to observe the distribution of count abundance across all Zebrafish (*Danio rerio)* and diet samples (*n* = 18) at a phylum level. The *y*-axis represents the fraction of samples, in which these individual amplicon sequence variants (ASVs) are present. The *x*-axis represents the average count abundance via a log scale. Each point represents an individual ASV point.

All bacteria discovered through QIIME2 (v2023.2) across all samples were displayed in Figure 1. The most abundant phyla seen across all samples were Proteobacteria, Firmicutes, Actinobacteriota, Planctomycetota, and Bacteroidota.

### Taxonomic distribution across diets

**FIGURE 2:**
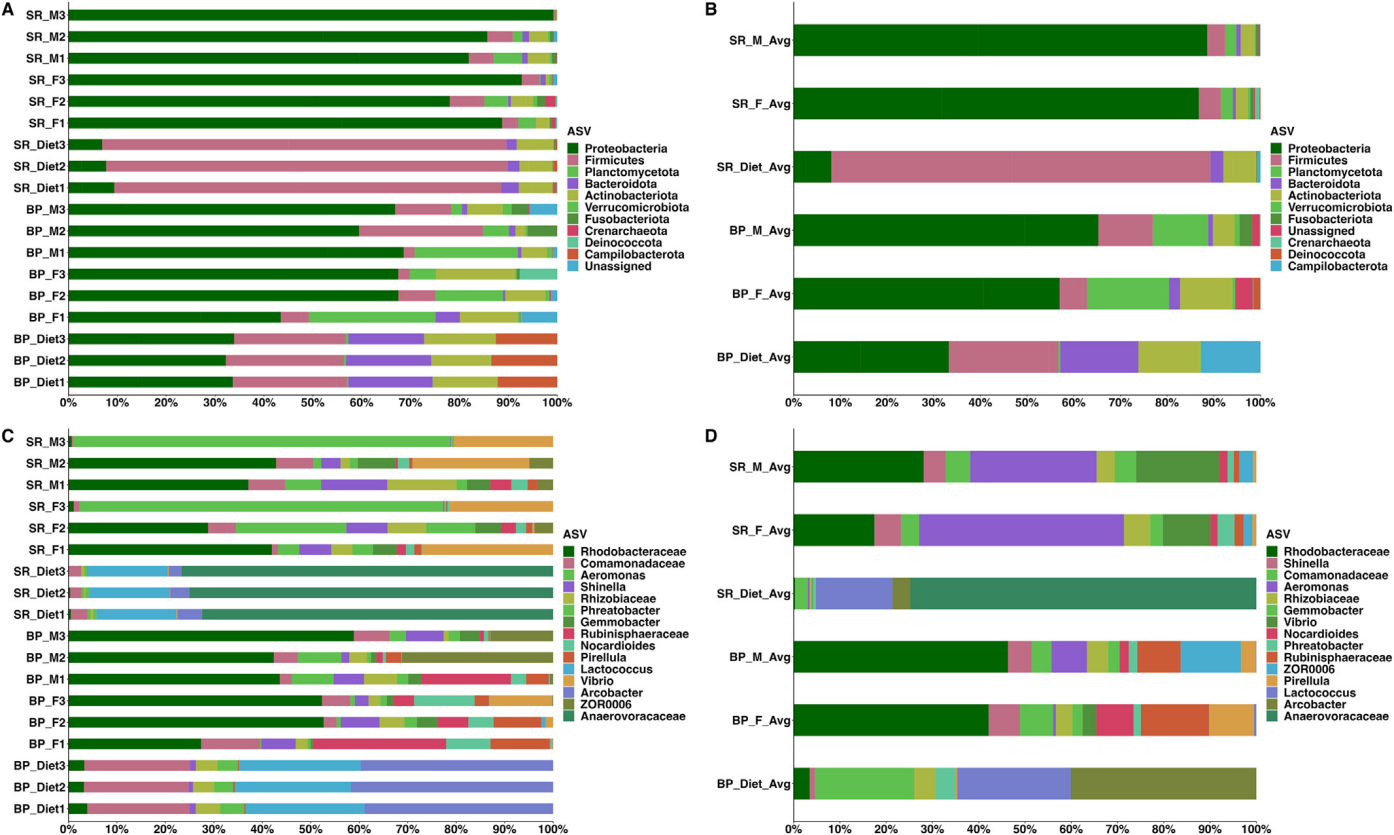
(A) The horizontal stacked column bar plot represents taxonomic abundance at the phylum level with the highest abundance across all *Danio rerio* samples (*n* = 18 total samples) and visualized using R (ggplot package). (B) Horizontal stacked column bar plot represents the mean phylum-level taxa across all samples. (C) Top 15 genus-level or the greatest available resolution of taxonomic rank across all samples (*n* = 18). (D) Horizontal stacked column bar plot represents the mean percentage of indicated taxa across all samples. The taxonomic identities were established utilizing the SILVA v138 (silva-138-99-nb-classifier.qza) database, determined by the Quantitative Insights into Microbial Ecology (QIIME_2, v2023.2). SR_M = standard reference (SR) diet-fed male; SR_F = SR diet-fed female; SR_Diet = SR diet; BP M = bacterial protein (BP) diet-fed male; BP F = BP diet-fed female; BP_Diet = BP diet; SR_M_Avg = the mean bacterial abundance of SR diet-fed males; SR_F_Avg = the mean bacterial abundance of the female fed with the SR diet; SR_Diet_Avg = the mean bacterial abundance of the SR diet; BP_M_Avg = the mean bacterial abundance of the BP diet-fed males; BP_F_Avg = the mean bacterial abundance of the BP diet-fed females; and BP_Diet_Avg = the mean bacterial abundance of the BP diet. A complete list of the output of QIIME2 (v2023.2) taxa and their abundances is presented in Supplemental Table 1.

Firmicutes were the dominant taxon in the SR diet, and in contrast, the BP diet contained an abundance of Proteobacteria and Firmicutes members as well as significant amounts of Actinobacteriota, Bacteroidota, and Campilobacterota (Table 3). The SR diet was dominated by *Anaerovoracaceae* (75%) and *Lactococcus* (∼17%). In comparison, the BP diet was primarily composed of *Arcobacter* (∼40.1%) and *Lactococcus* (∼24.5%). An abundance of Comamonadaceae (∼22%) was observed in the BP diet but was only present at low levels (∼3%) in the SR diet (Table 4 and Figure 2).

**TABLE 3:** Percentage of taxonomic distribution at the phylum level across samples

ASV	BP_Diet_Avg (%)	BP_F_Diet (%)	BP_M_Diet (%)	SR_Diet_Avg (%)	SR_F_Avg (%)	SR_M_Avg (%)
Proteobacteria	33.3	59.5	65.0	8.0	86.5	89.0
Firmicutes	23.5	5.2	13	81.2	4.6	3.6
Planctomycetota	0.4	15.0	9.5	0.0	2.8	2.6
Actinobacteriota	13.1	11.7	2.3	2.1	2.0	2.2
Bacteroidota	16.7	1.8	1.0	2.7	0.6	0.8
Campilobacterota	12.8	0.0	0.0	0.7	0.0	0.0
Unknown	0.0	2.8	2.1	0.0	0.3	0.2
Fusobacteriota	0.0	0.2	3.1	0.2	0.7	0.5
Actinobacteriota	0.2	0.4	2.5	5.0	0.7	0.7
Verrucomicrobiota	0.1	0.5	1.1	0.0	0.5	0.3
Deinococcota	0.0	2.7	0.1	0.0	0.2	0.0
Crenarchaeota	0.0	0.1	0.2	0.0	1.0	0.1

Shown are the top 12 phyla across the mean bacterial abundance of sample groups. Sample assignments are as follows: BP_Diet_Avg = mean of the BP diet; BP_F_Avg = mean BP-fed female *D. rerio*; BP_M_Avg = mean BP-fed male *Danio rerio*; SR_Diet_Avg = the mean of the SR diet; SR_F_Avg = mean SR-fed female *D. rerio*; SR_M_Avg = mean SR-fed male *D. rerio*.

**TABLE 4:** Highest resolution of taxa

ASV	BP_Diet_Avg	BP_F_Avg	BP_M_Avg	SR_Diet_Avg	SR_F_Avg	SR_M_Avg
Rhodobacteraceae	3.5	44.1	48.3	0.3	24.0	26.9
Aeromonas	0.0	0.7	7.0	0.4	34.1	29.0
Vibrio	0.0	5.0	0.1	0.2	16.3	14.8
Rubinisphaeraceae	0.0	12.7	6.9	0.0	1.7	1.6
Comamonadaceae	21.5	6.9	5.0	2.8	2.7	5.2
Shinella	1.1	6.0	5.3	0.0	5.1	6.0
ZOR0006	0.0	0.1	15.0	0.0	1.3	2.7
Rhizobiaceae	4.6	3.3	3.8	0.5	4.2	5.5
Pirellula	0.3	8.3	2.7	0.0	0.9	0.9
Nocardioides	0.0	8.9	1.6	0.0	1.4	1.9
Gemmobacter	0.0	2.0	2.6	0.0	3.4	4.2
Arcobacter	40.1	0.0	0.0	3.9	0.0	0.0
Anaerovoracaceae	0.0	0.0	0.0	74.7	0.0	0.0
Phreatobacter	4.4	1.5	1.8	0.7	4.8	1.4
Lactococcus	24.5	0.4	0.1	16.6	0.1	0.0

Shown are the top 12 genera or highest resolution of taxa outputted via QIIME2 (v2023.2), across the mean of sample groups. Sample assignments are as follows: Sample assignments are as follows: BP_Diet_Avg = mean of the BP diet; BP_F_Avg = mean BP-fed female *Danio rerio*; BP_M_Avg = mean BP-fed male *D. rerio*; SR_Diet_Avg = the mean of the SR diet; SR_F_Avg = mean SR-fed female *D. rerio*; SR_M_Avg = mean SR-fed male *D. rerio*.

### Taxonomic distribution across SR-fed and BP-fed *D. rerio* samples

*D. rerio* fed with either the BP or SR diet samples exhibited Proteobacteria as the dominant taxa. At the greatest possible taxonomic resolution outputted via QIIME2 (v2023.2), Rhodobacteraceae and *Aeromonas* were found to be highly abundant across all *D. rerio* fed with either the BP or SR diet (Figure 2). Female *D. rerio* fed with the SR diet was dominated via *Aeromonas* (∼34%), Rhodobacteraceae (∼24%), and *Vibrio* (∼16%). In contrast, female *D. rerio* fed with the BP diet was dominated via Rhodobacteraceae (∼44), Rubinisphaeraceae (13%), and *Nocardioides* (∼9%). Male *D. rerio* fed with the SR diet resulted in a microbial composition dominated via *Aeromonas* (∼29%), Rhodobacteraceae (∼26%), and *Vibrio* (∼15%). In contrast, male *D. rerio* fed with the BP diet resulted in a higher abundance of Rhodobacteraceae (∼48%) and Firmicutes member ZOR0006 (∼15%).

### Alpha diversity and beta diversity

**FIGURE 3:**
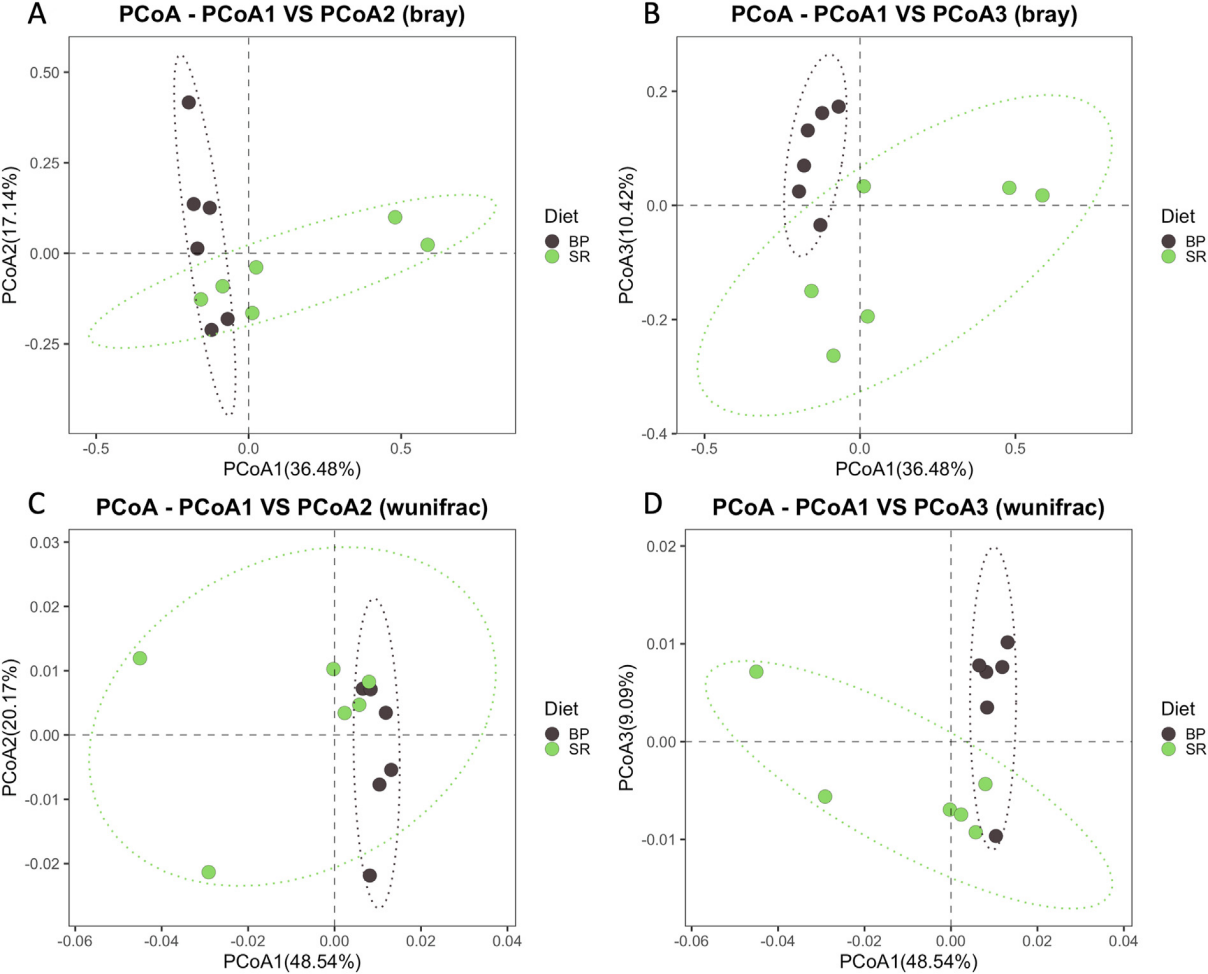
Beta diversity analysis of gut microbiota of *Danio rerio* was observed across all similarity metrics determined for the ASV table. Beta diversity metrics were determined via taxonomy outputted via QIIME2 (v2023.2), plotted utilizing R (MicrobiotaProcess v1.6.6). (A) Representation of PCoA1 against PCoA2 calculated using Bray–Curtis metrics. (B) Representation of PCoA1 against PCoA3 calculated using Bray–Curtis metrics. (C) Representation of PCoA1 against PCoA2 calculated using weighted UniFrac and PCoA1 against PCoA3 calculated using weighted UniFrac. Sample assignments are as follows: BP diet = bacterial protein diet (brown); BP = *Danio rerio* fed with the bacterial protein diet (gray); SR = *D. rerio* fed with the SR diet (green). Ellipses were added based off default settings in MicrobiotaProcess (v1.6.6) confidence = 0.9.

Beta diversity was determined utilizing Bray–Curtis and weighted UniFrac metrics across *D. rerio* samples. *D. rerio* fed with the BP diet displayed intrasample clustering within diet treatment (Figure 3); however, the *D. rerio* fed with the SR diet displayed intrasample variation within diet treatment (Figure 3). PERMANOVA statistics were tested against all *D. rerio* samples and supported significant dissimilarity among sample groups (*R*^2^ = 0.23), with *P* values (<0.05). PERMDISP revealed no significant dispersion of samples (*P* > 0.05).

**FIGURE 4:**
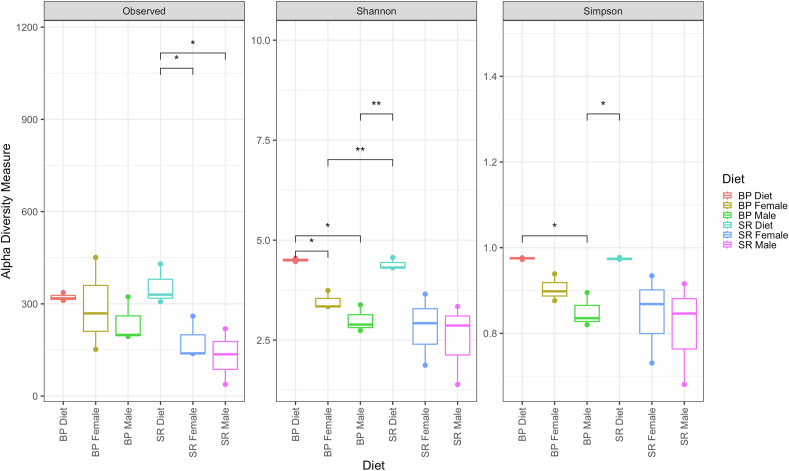
Alfa-diversity measurements (observed ASVs, Shannon diversity index, and the Simpson’s index) across all sample groups, based off QIIME2 (v2023.2) output and plotted using phyloseq (v1.38.0). Sample assignments are as follows: BP diet = bacterial protein diet; BP female = female *Danio rerio* fed with the bacterial protein diet; BP male = male *D. rerio* fed with the bacterial protein diet; SR diet = standard reference diet; SR female = female *D. rerio* fed with the standard reference diet; SR male = male *D. rerio* fed with the standard reference diet. ∗*P* value < 0.05, ∗∗*P* value < 0.005.

Alfa diversity observed ASVs revealed no significant (*P* > 0.05) difference between BP diet, and BP-fed male, and BP-fed female groups; however, a significant (*P* <0.05) difference between SR diet, and SR-fed female, and SR-fed male was observed. Shannon diversity revealed a significant difference (*P* < 0.05) between BP diet, BP-fed female, and BP-fed male samples. A significant difference (*P* < 0.05) was observed between the BP-fed female sample group and the SR diet; likewise, a significant difference (*P* < 0.05) was observed between the BP-fed male sample group and the SR diet. Simpson diversity revealed a significant difference between the BP diet and BP-fed male sample groups. Additionally, a significant difference was observed between BP-fed males, and the SR diet (Table 5 and Figure 4).

**TABLE 5:** Alfa diversity

Sample_ID	Observed	Shannon	Simpson
BP_Diet1	337	4.54801025	0.97634585
BP_Diet2	318	4.46317514	0.97251731
BP_Diet3	311	4.49745506	0.97537932
BP_F1	451	3.74158284	0.93894371
BP_F2	269	3.3393815	0.87626449
BP_F3	152	3.34374512	0.89825284
BP_M1	323	3.38358116	0.89523321
BP_M2	194	2.74040476	0.83548435
BP_M3	199	2.88856506	0.82016929
SR_Diet1	430	4.56789888	0.97728218
SR_Diet2	330	4.31470472	0.97290372
SR_Diet3	307	4.30734538	0.97374749
SR_F1	138	2.92395894	0.86846615
SR_F2	260	3.65057579	0.93459469
SR_F3	139	1.87271695	0.73083181
SR_M1	136	3.34267221	0.91578715
SR_M2	219	2.86554469	0.84633826
SR_M3	38	1.39260246	0.68093423

Alfa diversity was determined utilizing observed number of ASVs, Shannon diversity index, and the Simpson diversity index, across diet and *Danio rerio* samples.

### Differential expression analysis of microbial communities in *D. rerio* fed BP and SR

**FIGURE 5:**
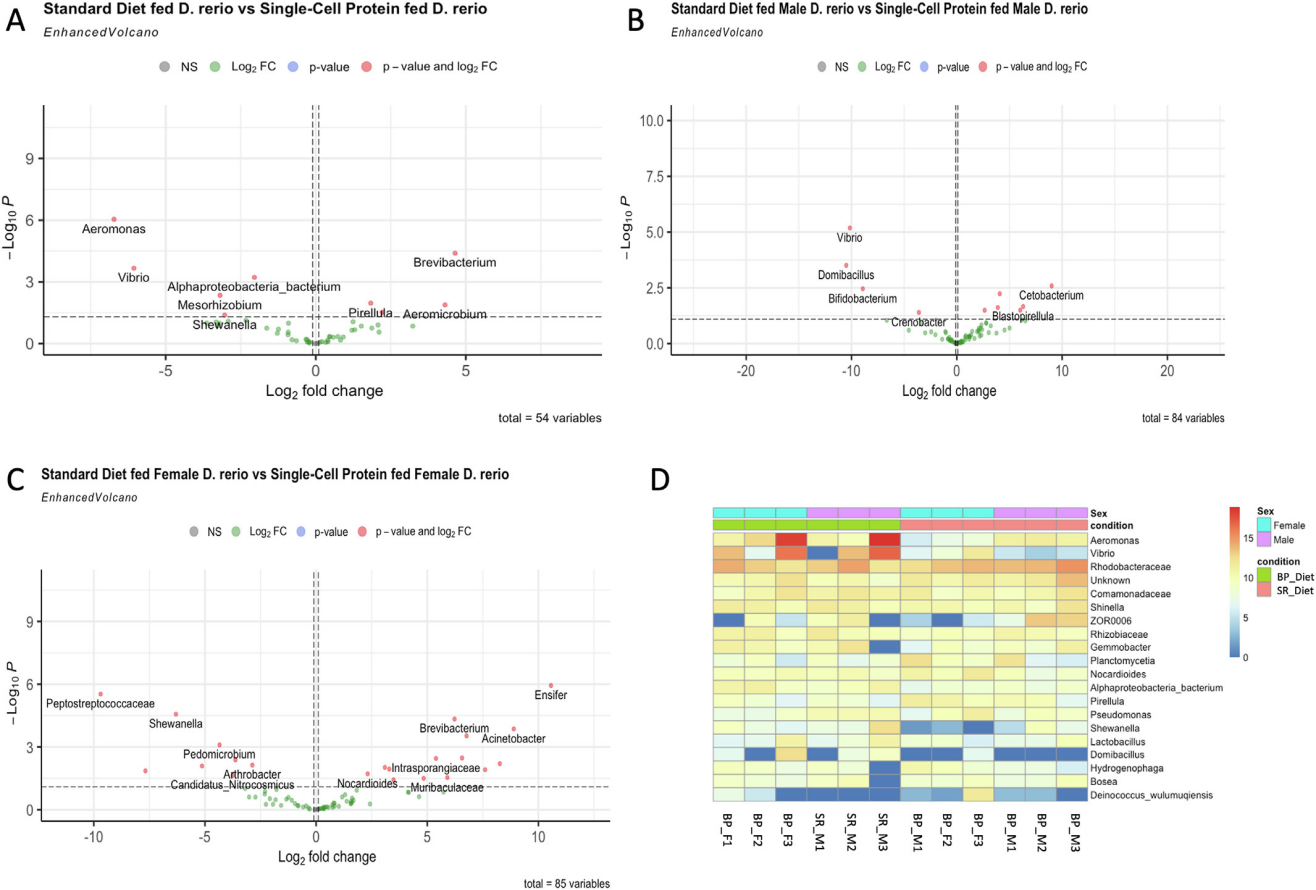
Differential expression analysis was utilized to determine taxonomic differences between *Danio rerio* fed with the standard reference diet and *D. rerio* fed with the bacterial protein diet determined via DeSeq2 (v1.34.0). The standard reference diet was utilized as the control. Volcano plots were determined via DeSeq2 parameters set to display *P* value ≤ 0.05, and fold change ≥ 0.5. Red points display significance (*P* < 0.05). The log2foldchange represents the *x*-axis and –log10*p*value represents the *y*-axis. Gray points represent nonsignificant taxa, green points represent a Log2 fold change > 0.5 or < −0.5, blue points represent a significance *P* value (*P* ≤ 0.05), and red points indicate a significant *p* value (≤0.05) and log2foldchange > 0.5 or < −0.5). (A) A volcano plot based off the output of DeSeq2 analysis across all *D. rerio* samples. (B) A volcano plot based off the output of DeSeq2 analysis across all male *D. rerio* samples. (C) A volcano plot based off the output of DeSeq2 analysis across all female *D. rerio* samples. (D) A heatmap displays base mean of bacteria present in samples, and color scale represents base means value (blue = low, red = high).

DeSeq2 analysis revealed a significantly higher expression of *Brevibacterium*, and lower expression of *Aeromonas* in *D. rerio* fed with the BP diet in contrast to the SR diet across all samples (Figure 5A). A significant expression of *Cetobacterium* and a lower expression of *Vibrio* were observed in male *D. rerio* fed with the BP diet, in contrast to the male *D. rerio* fed with the SR diet (Figure 5B). A significant expression of *Ensifer* and lower expression of Peptostreptococcaceae was observed in female *D. rerio* fed with the BP diet, in contrast to the male *D. rerio* fed with the SR diet (Figure 5C).

### Copresence, co-exclusion, and key taxa in single-cell and standard diets

**FIGURE 6:**
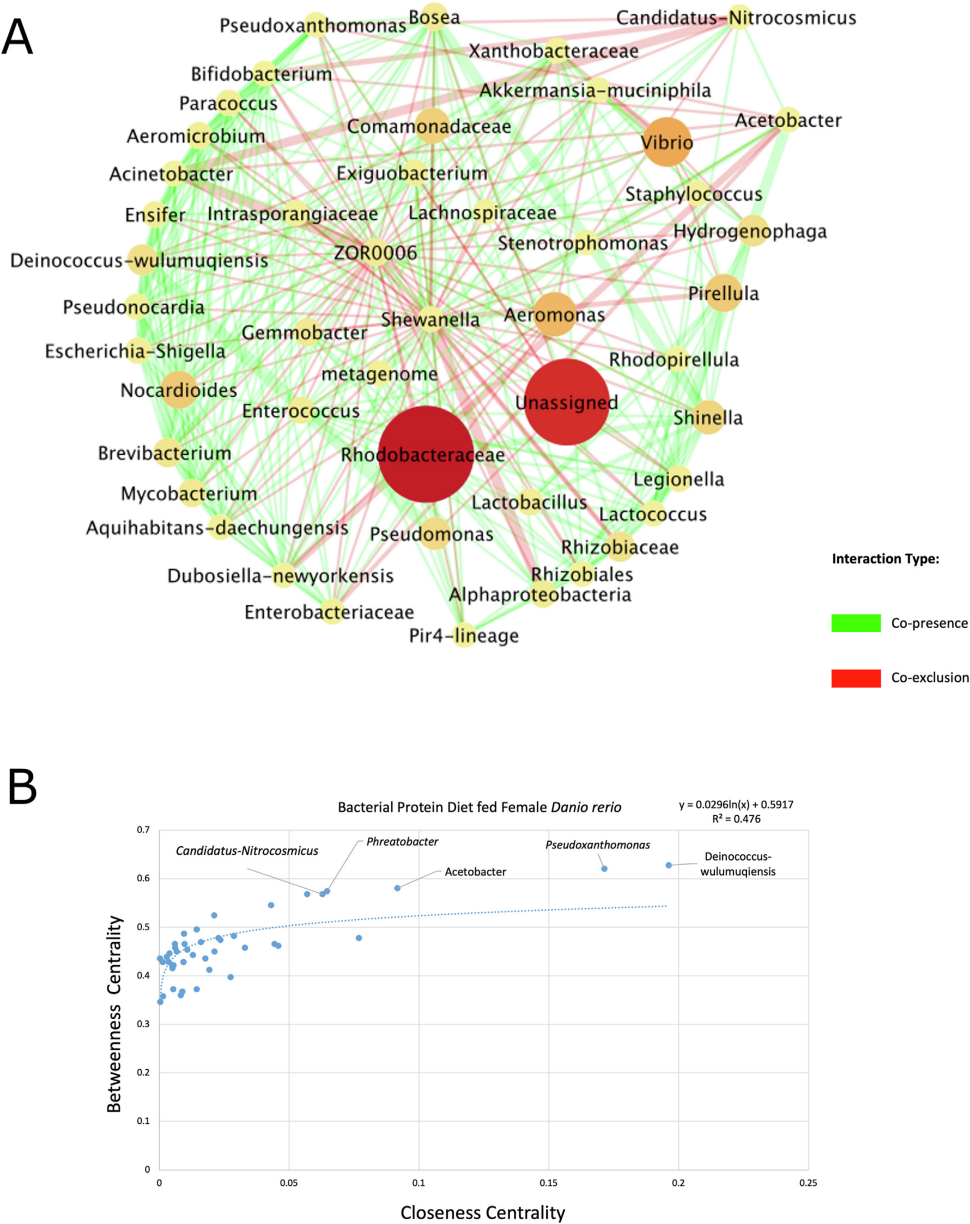
(A) The network represents the gut ecosystem of female *D. rerio* fed the bacterial protein. The co-occurrence patterns and interactions were established via taxonomic entries established previously in QIIME2 (v2023.2) and were selected for analysis by Co-occurrence network inference (CoNet v1.1.1) and Cytoscape (v3.8.3); 2/3 of samples showing a non–zero value entry were used against taxonomic entries with the cumulative row sum of 200, following ensemble approach, which includes Kullback–Leibler, Bray–Curtis, Pearson, and Spearman, and mutual information metrics. The merge via union method was chosen to merge the top 200 and bottom 200 edges. The final CoNet (v1.1.1) network analysis displayed, displays edges represented via *q* value (the weight of lines indicated this). The edges displayed are coded as green (indicating copresence) and red (indicating co-exclusion). The nodes represent taxa identified as part of the network, which were scaled via size and color according to abundance. The final network was displayed using the default layout in Cytoscape (v3.8.3). The radial layout was not applicable due to the large degree and associations in the network. (B) The scatter plot represents the output of NetworkAnalyzer (v2.7), which provided topologic networks, and were inputted into Microsoft Excel Software to demonstrate potential patterns of key (keystone) taxon between the taxonomic entries outputted via QIIME2 (v.2023.2) of the female *D. rerio* fed with the bacterial protein diet based off closeness and betweenness centrality, this was also placed in descending order via degree (number of copresence and co-exclusion edges). The linear regression analysis was established using Microsoft Excel Software. Linear regression between closeness and betweenness centrality was displayed on the plot as a logarithmic line (*R*^2^ value = 0.476).

The network of the gut ecosystem of female *D. rerio* fed with the BP diet CoNet yielded 55 nodes and 366 edges (Figure 6A). The network properties were identified utilizing the NetworkAnalyzer (v2.7) tool, and outputted the average number of neighbors of 13.3, the characteristic path length of 2.045, with a network density of 0.123, and a clustering coefficient of 0.353. *Deinococcus wulumuqiensis* resulted in the largest degree (23 total), displaying co-exclusion for most of these associations (16 total). The closeness centrality values were plotted against betweenness centrality values to present trends via scatter plot analysis (Figure 6B). The top 5 candidate key taxa in *D. rerio* fed with the SCP female were selected utilizing Berry and Widder's [49] topologic qualities of taxonomic node descriptions and were further ranked by their closeness centrality. The top 5 candidate key taxa were *Deinococcus wulumuqiensis*, *Acetobacter*, *Phreatobacter*, *Pseudoxanthomonas*, and *Candidatus nitrocosmicus* (Figure 6B).

**FIGURE 7:**
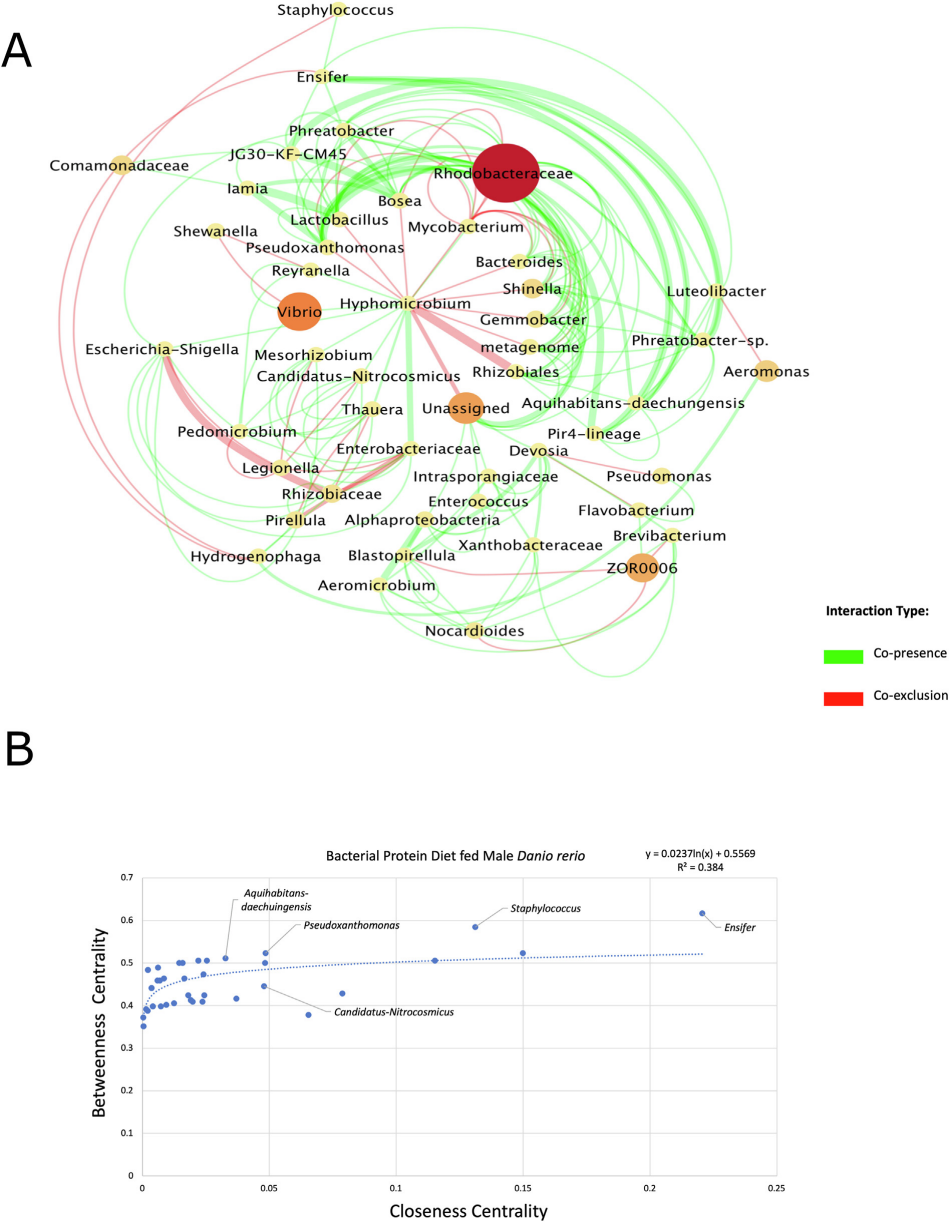
(A) The network represents the gut ecosystem of male *Danio rerio* fed with a bacterial protein diet. The co-occurrence patterns and interactions were established via taxonomic entries established previously in QIIME2 (v2023.2) and were selected for analysis by Co-occurrence network inference (CoNet v1.1.1) and Cytoscape (v3.8.3); 2/3 of samples showing a nonzero value entry were used against taxonomic entries with the cumulative row sum of 200, following ensemble approach, which includes Kullback–Leibler, Bray–Curtis, Pearson, and Spearman, and mutual information metrics. The merge via union method was chosen to merge the top 200 and bottom 200 edges. The final CoNet (v1.1.1) network analysis displayed, displays edges represented via *q* value (the weight of lines indicated this). The edges displayed are coded as green (indicating copresence) and red (indicating co-exclusion). The nodes represent taxa identified as part of the network, which were scaled via size and color according to abundance. The final network was displayed using the radial layout from yFiles plugin (v1.0) in Cytoscape (v3.8.3). (B) The scatter plot represents the output of NetworkAnalyzer (v2.7), which provided topologic networks, and were inputted into Microsoft Excel Software to demonstrate potential patterns of key (keystone) taxon between the taxonomic entries outputted via QIIME2 (v.2023.2) of the single-cell protein diet-fed male *D. rerio* based off closeness and betweenness centrality, this was also placed in descending order via degree (number of copresence and co-exclusion edges). The linear regression analysis was established using Microsoft Excel Software. Linear regression between closeness and betweenness centrality was displayed on the plot as a logarithmic line (*R*^2^ value = 0.384).

The gut ecosystem network of male *D. rerio* fed with the BP diet yielded 46 nodes and 273 edges (Figure 7A). The network properties were identified utilizing the NetworkAnalyzer (v2.7) tool, and outputted the average number of neighbors of 11.9, the characteristic path length of 1.948, with a network density of 0.132, and a clustering coefficient of 0.372. The taxon *Ensifer* resulted in the largest degree (22 total), displaying copresence for most of these associations (13 total). The closeness centrality values were plotted against betweenness centrality values to present the potential trends via a scatter plot analysis (Figure 7B). The top 5 candidate key taxa were *Ensifer*, *Staphylococcus*, *Pseudoxanthomonas*, *Aquihabitans daechuingensis*, and *Candidatus nitrocosmicus* (Figure 7B).

**FIGURE 8:**
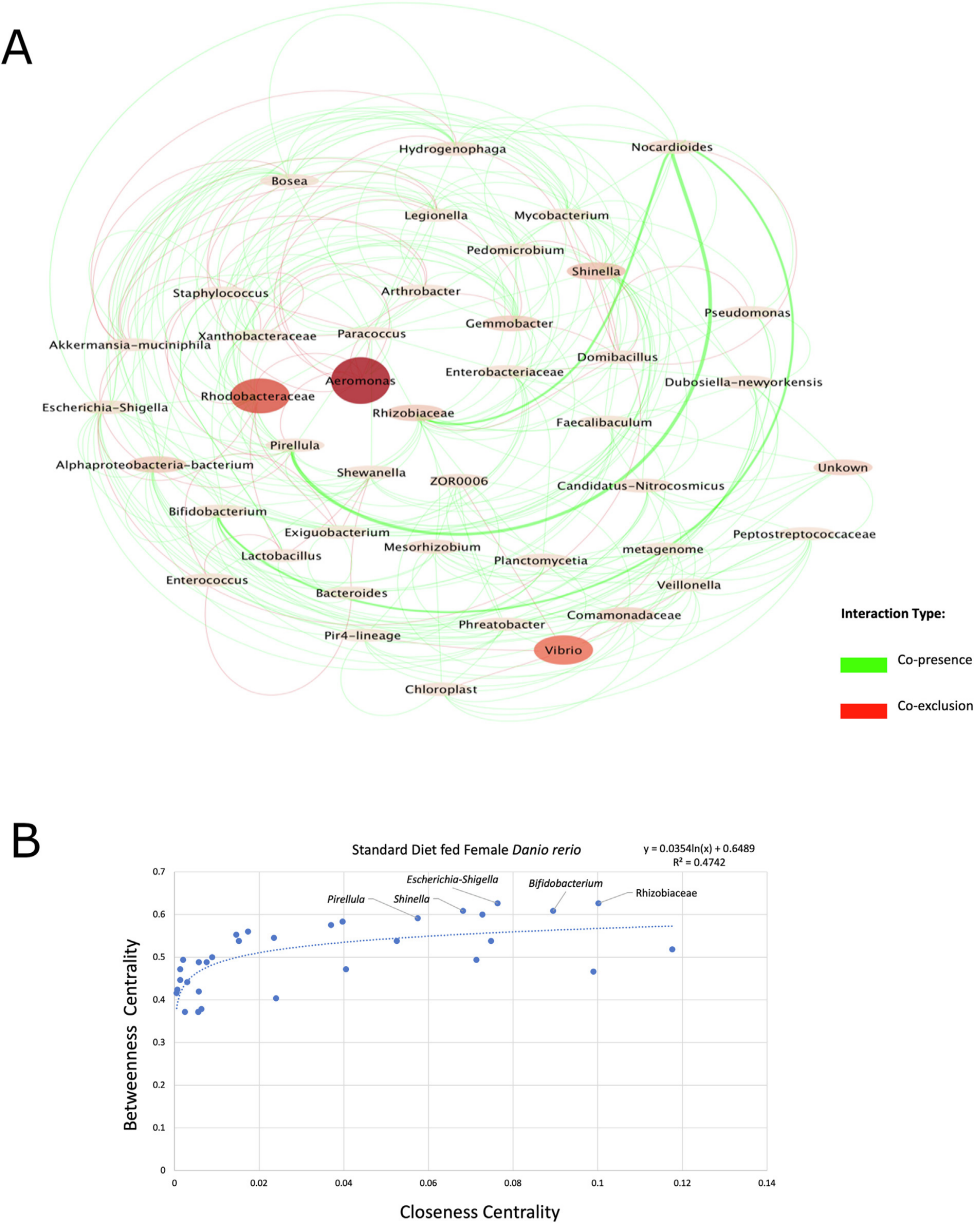
(A) The network represents the gut ecosystem of female *Danio rerio* fed with the standard reference diet. The co-occurrence patterns and interactions were established via taxonomic entries established previously in QIIME2 (v2023.2) and were selected for analysis by Co-occurrence network inference (CoNet v1.1.1) and Cytoscape (v3.8.3); 2/3 of samples showing a nonzero value entry were used against taxonomic entries with the cumulative row sum of 200, following ensemble approach, which includes Kullback–Leibler, Bray–Curtis, Pearson, and Spearman, and mutual information metrics. The merge via union method was chosen to merge the top 200 and bottom 200 edges. The final CoNet (v1.1.1) network analysis displayed, displays edges represented via *q* value (the weight of lines indicated this). The edges displayed are coded as green (indicating copresence) and red (indicating co-exclusion). The nodes represent taxa identified as part of the network, which were scaled via size and color according to abundance. The final network was displayed using the yFiles plugin, and the radial layout (v1.0) in Cytoscape (v3.8.3). (B) The scatter plot represents the output of NetworkAnalyzer (v2.7), which provided topologic networks, and were inputted into Microsoft Excel Software to demonstrate potential patterns of key (keystone) taxon between the taxonomic entries outputted via QIIME2 (v.2023.2) of the female *D. rerio* fed with a standard diet based off closeness and betweenness centrality, this was also placed in descending order via degree (number of copresence and co-exclusion edges). The linear regression analysis was established using Microsoft Excel Software. Linear regression between closeness and betweenness centrality was displayed on the plot as a logarithmic line (*R*^*2*^ value = 0.4742).

The gut ecosystem network of female *D. rerio* fed with the SR diet yielded 46 nodes and 273 edges (Figure 8A). The network properties were identified utilizing the NetworkAnalyzer (v2.7) tool, and outputted the average number of neighbors of 13.070, the characteristic path length of 2.164, with a network density of 0.311, and a clustering coefficient of 0.750. The bacterium with the largest degree was Rhizobiaceae (22 total), displaying copresence for most of these associations (21 total). The closeness centrality values were plotted against betweenness centrality values to present established trends via scatter plot analysis (Figure 8B). The top 5 candidate key taxa were Rhizobiaceae, *Shinella*, *Pirellula, Escherichia-Shigella*, and *Bifidobacterium* (Figure 8B).

**FIGURE 9:**
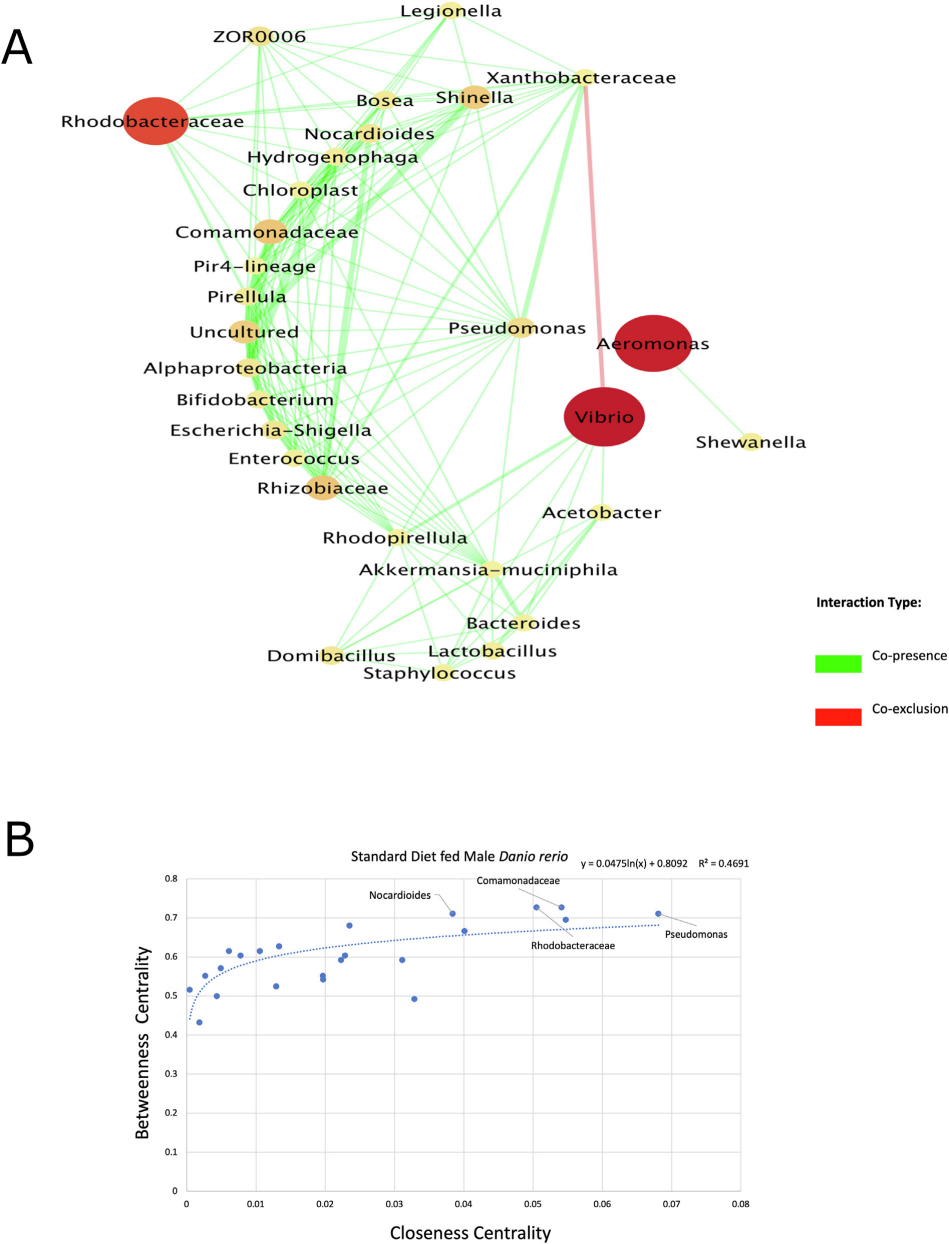
(A) The network represents the gut ecosystem of male *Danio rerio* fed with the standard diet. The co-occurrence patterns and interactions were established via taxonomic entries established previously in QIIME2 (v2023.2) and were selected for analysis by Co-occurrence network inference (CoNet v1.1.1) and Cytoscape (v3.8.3); 2/3 of samples showing a nonzero value entry were used against taxonomic entries with the cumulative row sum of 200, following ensemble approach, which includes Kullback–Leibler, Bray–Curtis, Pearson, and Spearman, and mutual information metrics. The merge via union method was chosen to merge the top 200 and bottom 200 edges. The final CoNet (v1.1.1) network analysis displayed, displays edges represented via *q* value (the weight of lines indicated this). The edges displayed are coded as green (indicating copresence) and red (indicating co-exclusion). The nodes represent taxa identified as part of the network, which were scaled via size and color according to abundance. The final network was displayed using the yFiles plugin, and the radial layout (v1.0) in Cytoscape (v3.8.3). (B) The scatter plot represents the output of NetworkAnalyzer (v2.7), which provided topologic networks, and were inputted into Microsoft Excel Software to demonstrate potential patterns of key (keystone) taxon between the taxonomic entries outputted via QIIME2 (v.2023.2) of the male *D. rerio* fed with the standard reference diet based off closeness and betweenness centrality, this was also placed in descending order via degree (number of copresence and co-exclusion edges). The linear regression analysis was established using Microsoft Excel Software. Linear regression between closeness and betweenness centrality was displayed on the plot as a logarithmic line (*R*^2^ value = 0.504).

The network of the male *D. rerio* fed with the SR diet yielded 33 nodes and 245 edges (Figure 9A). The network properties were identified utilizing the NetworkAnalyzer (v2.7) tool, and outputted the average number of neighbors of 14.8, the characteristic path length of 1.689, with a network density of 0.462, and a clustering coefficient of 0.778. The largest abundance of bacteria was Rhodobacteraceae (21 total), displaying copresence for most of these associations (21 total). The closeness centrality values were plotted against betweenness centrality values to present established trends via scatter plot analysis (Figure 9B). The top 4 candidate key taxa were Rhodobacteraceae, Comamonadaceae, *Nocardioides*, and *Pseudomonas* (Figure 9B). In the top 5 established, one key taxon was unidentified via QIIME2 (v2023.2).

### Predicted functional analysis

**FIGURE 10:**
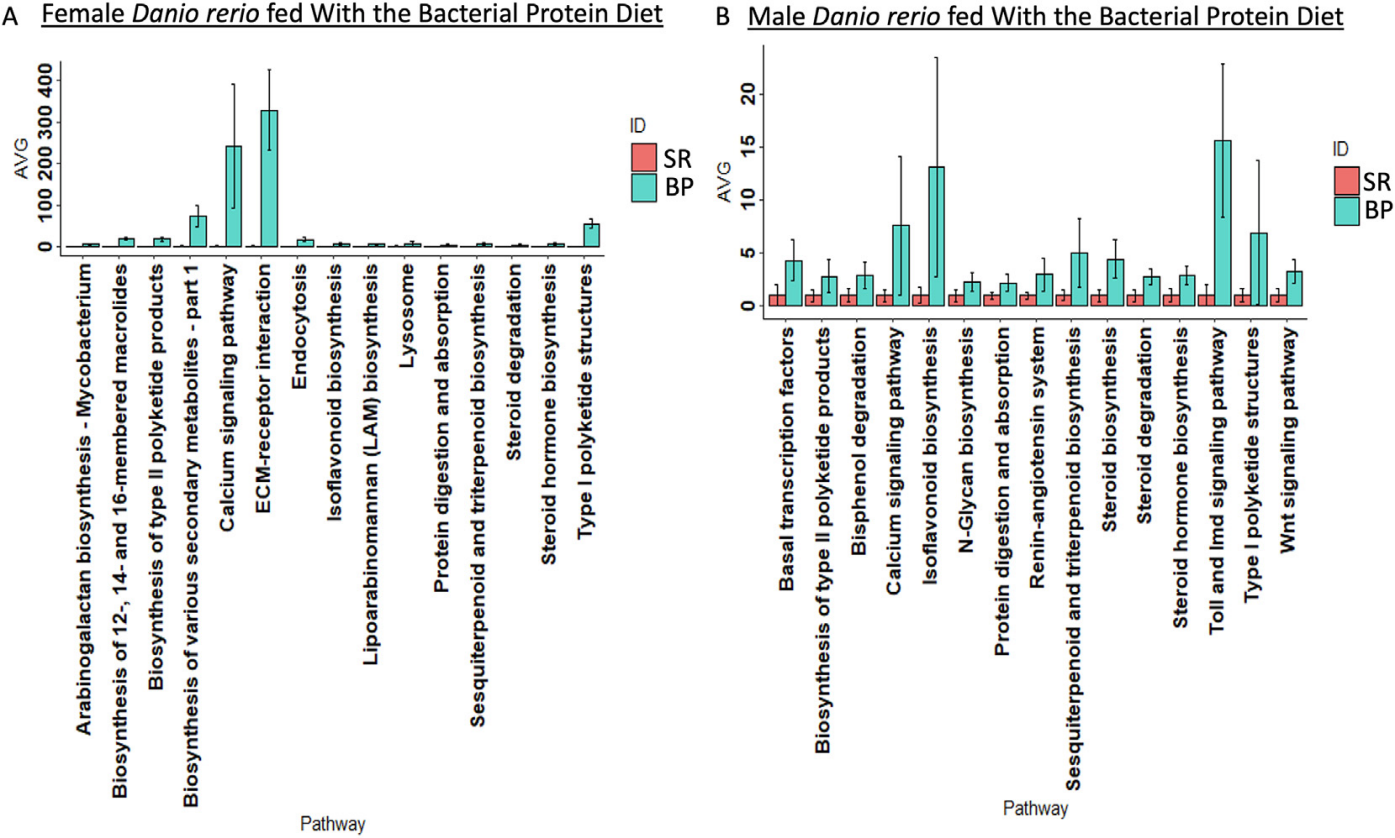
Vertical barplot represents the top 15 pathways outputted via PICRUSt2 (v.2.5.2) at a KEGG level 3 manually curated mapfile from https://www.genome.jp/kegg-bin/get_htext?ko00001.keg (accessed on 4 April 2023), based off the output off QIIME2 (v2023.2). The values outputted via PICRUSt2 (v2.5.2) were normalized to 1 based off the standard reference diet, and the average was represented (*y*-axis). (A) Representation of female *Danio rerio* variations in functional pathway abundance. (B) Representation of male *D. rerio* variations in functional pathway abundance. Sample assignments are as follows: *D. rerio* fed with the standard reference diet labeled as SR, *n* = 3 (red bars); *D. rerio* fed with the bacterial protein diet labeled as BP, *n* = 3 (blue bars). Error bars represent standard error of the mean.

**FIGURE 11:**
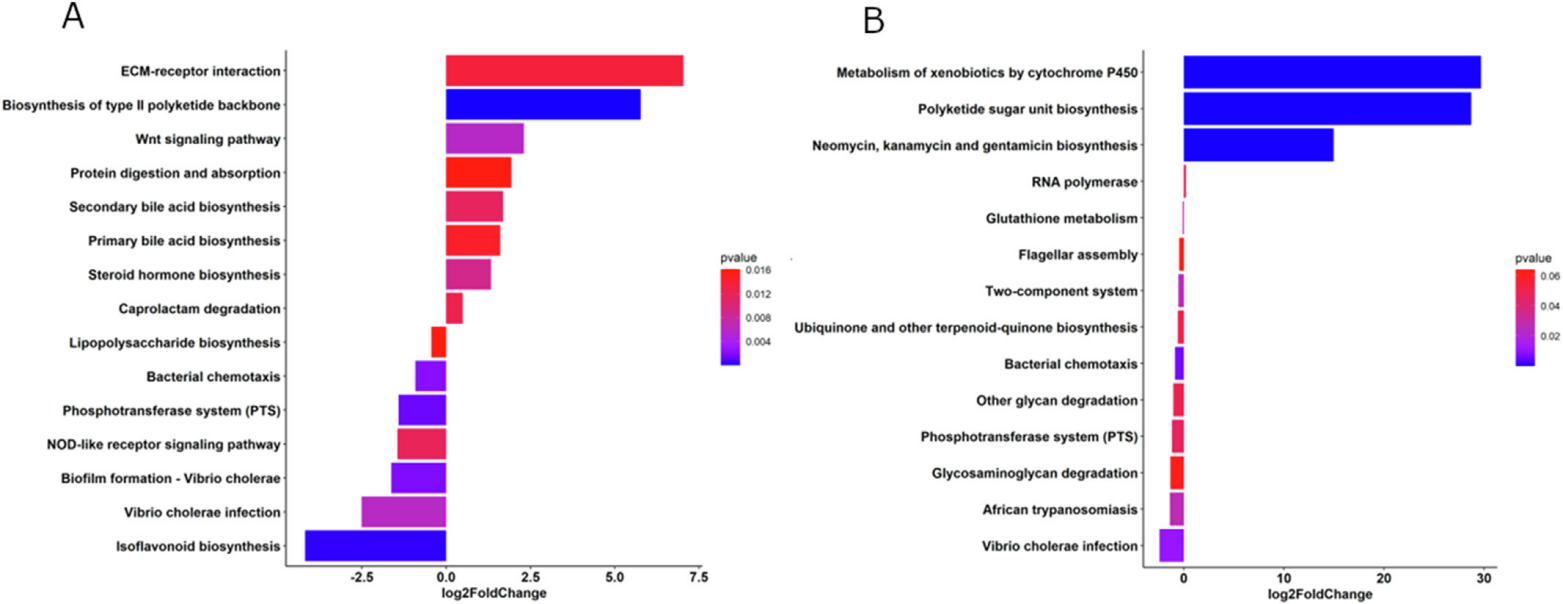
The horizontal divergent barplot represents the analysis of the KEGG level 3 pathways using *D. rerio* fed with the standard reference diet as a control. The top 16 pathways were selected based off *p* value (*P* < 0.05). The functional categories were determined via PICRUSt2 (v2.3.0-b) script pathway_pipeline.py with a manually curated mapfile from https://www.genome.jp/kegg-bin/get_htext?ko00001.keg (accessed on 4 April 2023). Blue bars represent downregulated pathways in *D. rerio* fed with the bacterial protein, and red bars represents upregulated pathways in the bacterial protein diet. (A) Representation of female *D. rerio* fed with the bacterial protein diet. (B) Representation of male *D. rerio* fed with the bacterial protein diet.

The Nearest Sequenced Taxon Index values calculated through PICRUST (v2.3.0) showed an average value of 0.046 (ranging from 0.01–0.07) (data not shown). Female *D. rerio* fed with the BP diet resulted in a significant upregulation (*P* < 0.05) in KEGG pathways: The extracellular matrix (ECM)–receptor interaction and calcium signaling pathway (Figure 10A). The differential functional analysis of these samples resulted in an upregulation of pathways: Biosynthesis of type II polyketide backbone, Wingless-related integration site (Wnt) signaling pathway, protein digestion and absorption, secondary bile acid biosynthesis, primary bile acid synthesis, and steroid hormone biosynthesis (Figure 11A). Male *D. rerio* fed with the BP diet resulted in an upregulation in the calcium signaling pathway, isoflavonoids biosynthesis, Toll and Imd signaling pathway, and type 1 polyketide structure (Figure 11A). Differential expression analysis resulted in a significant upregulation of KEGG pathways metabolism of xenobiotics by cytochrome p450 and polyketide sugar unit biosynthesis (Figure 11B).

## Discussion

Williams et al. [4] reported that consumption of the BP diet led to comparable weight gain to *D. rerio* fed the SR diet but also resulted in significantly less adiposity in females and invoked the expression of a transcriptome showing significant changes in liver gene ontologies related to lipid and cholesterol metabolism. The microbiome data in the current study were derived from gut samples collected from the *D. rerio* used in that study, and thus directly comparable with the physiologic and transcriptome data reported in the study by Williams et al [4].

Samples of the SR and BP diets revealed distinct differences in endogenous bacterial communities. The majority of bacterium species present in diet samples were not found to colonize the intestinal tract of *D. rerio* with the exceptions of Rhodobacteraceae and Comamonadaceae. Comamonadaceae was seen across all diet and gut sample groups; however, there may be potential limitations in detection due to representatives of less-abundant bacteria not being collected during the sample preparation. *D. rerio* fed with the BP diet displayed differences in gut microbial composition to *D. rerio* fed with the SR diet. Undetected microbes present in the diet may have some influence on the ratio of resident microbiota found in the 16s rRNA sequencing of the *D. rerio* gut. Further investigation is required to understand the potential effect these low-abundant bacteria may have on the *D. rerio* microbiome.

Williams et al. [4] reported reduced fat deposition in females and, to a lesser extent, in male *D. rerio* fed the BP diet. We predict that low adiposity is associated with the relative abundance of Rhodobacteraceae present in the *D. rerio* fed with the BP diet compared with *D. rerio* fed the SR diet. Rhodobacteraceae members have been reported previously to aid in the binding of bile acids to cholesterol, thus potentially inhibiting micelle formation [54,55]. Rhodobacteraceae is a core family associated with the *D. rerio* microbiome [56] and was observed in both the BP diet and in the gut of *D. rerio* fed with the BP diet. In a related study, an abundance of Rhodobacteraceae was observed in the gut of *D. rerio* fed a low-fat diet, which also resulted in low adiposity [21].

There have been previous reports revealing a positive correlation between Comamonadaceae members to weight loss; however, *D. rerio* fed with the BP diet did not experience weight loss compared with *D. rerio* fed with the SR diet but did exhibit reduced fat deposition [57]. The family Comamonadaceae was observed in the BP diet as well as in the gut of male and female *D. rerio* fed with the BP diet. The large abundance of Comamonadaceae in the BP diet may contribute to changes in body composition; however, additional research will be needed to evaluate the role of Comamonadaceae members in lipid metabolism.

Microbial communities in the gut are typically variable among individual *D. rerio* outside of a core microbiome [58]. The variation in gut microbial populations in *D. rerio* fed the SR diet may be due in part to the presence of *Vibrio* and *Aeromonas*. *Vibrio* species can exclude *Aeromonas* spp., as *Vibrio* has been shown previously to have a competitive advantage [59,60]. In this study, abundances of *Vibrio* and *Aeromonas* varied among individuals in *D. rerio* fed with the SR diet. In contrast, *D. rerio* fed with the BP protein diet revealed distinct clustering among individuals, with distinct contributions by members of the family Rhodobacteraceae. Rhodobacteraceae members have been utilized as probiotics in aquatic models, usually in response to antibiotic treatments, to recolonize the gut with beneficial microbes [61]. *Phaeobacter* is a member of Rhodobacteraceae and has shown positive results in aiding microbiome colonization after antibiotic treatment, as well as being an inhibitor of *Vibrio* growth, and it is used typically to stop opportunistic pathogens from colonizing in finned fish [61]. The low abundance of Rhodobacteraceae coupled with the high and variable abundance of *Vibrio* may collectively contribute to the variable populations of endogenous microbes in the guts of *D. rerio* fed the SR diet. Given the differences in microbial communities present in the diets, and the concomitant difference in the BP- and SR-fed *D. rerio* gut microbiomes, particularly with regard to Rhodobacteraceae, further study of dietary microbes may provide insight into the development of formulations that can reduce microbial variability among gut samples.

Co-occurrence network analysis provided insight into interactions occurring among members of each of the gut microbial communities associated with males or females fed the BP or SR diets, as well as identifying potential key microbial members in those microbiomes. One such member, *Deinococcus wulumuqiensis,* was selected as the key taxon within the network in female *D. rerio* fed the BP diet. *Deinococcus* members have been linked to a distinct class of a quorum-sensing system, and in manipulating proteins in other gram-negative bacteria to utilize as an energy source [62,63]. *D*. *wulumuqiensis* was observed at a low-relative abundance inside the *D. rerio* gut; however, a potential role of this bacterium could be associated with a colonization resistance mechanism as the majority of interactions with *Deinococcus* were negative (co-exclusion) [38]. Further research is required to develop an understanding of the role of *D. wulumuqiensis* in *D. rerio*.

In contrast, co-occurrence network analysis labeled Rhizobiaceae as a key taxon in female *D. rerio* fed the SR diet. Rhizobiaceae was represented at a low abundance in the SR diet and female *D. rerio* fed with the SR diet and be part of the core microbiota in *D*. *rerio* [56]*.* The majority of this class functions in nitrogen fixation, but some members can metabolize potential toxic molecules [64]. It is reasonable to predict that this bacterium, although found in relatively low abundance, will exhibit a high degree of interaction with other taxa as many microbial members of the *D. rerio* gut microbiome are involved in nitrogen fixation.

*Ensifer* was a key taxon in male *D. rerio* fed with a BP diet. *Ensifer* has produced beneficial effects in plants and this microbe is known as a nitrogen-fixing bacteria [65]. Although found in relatively low abundance, Ensifer may potentially contribute to nitrogen fixation within the *D. rerio* gut ecosystem.

As noted previously, male *D. rerio* fed with the SR diet revealed Rhodobacteraceae as a key taxon, and members of the Rhodobacteraceae family are considered part of the core microbiota inhabiting *D*. *rerio*. Here, Rhodobacteraceae were revealed to have mostly positive associations with microbiota in the co-occurrence network. Rhodobacteraceae members utilize various inorganic and organic compounds as energy and are known for the metabolism of sulfur oxidation, production of secondary metabolites, and carbon monoxide oxidation [66,67]. In addition to their use as probiotics following antibiotic treatment, Rhodobacteraceae have been used as supplements in aquaculture for their potentially beneficial effect on growth outcomes. A member of Rhodobacteraceae, *Rhodobacter sphaeroides* protein was fed to *Litopenaeus vannamei* and increased growth performance [68]. Additionally, previous work in our laboratory has shown Rhodobacteraceae to be associated with a positive growth outcome in *D. rerio* [21]. Future research with this multispecies bacterial diet can identify beneficial strains, particularly from the Rhodobacteraceae family, and link beneficial microbial metabolites to growth outcomes.

Williams et al. [4] utilized global RNA sequencing and revealed shifts in cholesterol metabolism in the liver of female *D. rerio* fed a BP diet. Predictive functional pathway analysis, in combination with differential functional analysis, revealed KEGG pathways associated with primary and secondary bile acid synthesis in the gut of female *D. rerio* fed a BP diet. Bile acids go through modification via the host and microbial enzymes. Microbial modification of bile acids is observed across various vertebrates, including *D. rerio* [69]. Bacterial bile salt hydrolase has been observed to mediate microbe–host interaction, which can regulate host lipid metabolism. Microbe–host interactions have been shown to potentially contribute to cholesterol metabolism and weight gain [69]. Bile acid metabolites produced by, or modified by, the microbiota in *D. rerio* may be related to the observed differences in adiposity in *D. rerio* fed with the BP diet compared with the SR diet. Future studies can target specific bile acids associated with the microbiota to validate this hypothesis.

An additional observation of Williams et al. [4] was that calcium signaling was upregulated across female *D. rerio* fed a BP diet. Probiotics have been shown to mediate calcium signaling [70]. However, the extent of microbial involvement in calcium signaling in *D. rerio* metabolic homeostasis is not known.

The SR diet is a formulated diet designed to provide essential macro-/micronutrients for the development, growth, and reproduction of *D. rerio*. The unique shifts in microbial composition and functional profiles from *D. rerio* fed the SR diet to *D. rerio* fed the BP diet provide a key insight into the usage of a bacterial protein source as not only an alternative protein source but potentially as a preferred protein source. Williams et al. [4] revealed a maintenance of body weight, concomitant with a reduction in fat deposition, and beneficial changes in gene expression associated with cholesterol and lipid metabolism. Analysis of the gut microbiome revealed many pathways associated with lipid metabolism (steroid hormone synthesis and bile acid pathways). The extent and mechanism by which these specific gut microbes are contributing to host health are limited due to resolution restraints; however, our results coupled with the outcomes reported by Williams et al. [4] indicate that further study is merited. We hypothesize that the beneficial effects of the BP diet are due to the abundance of members of Rhodobacteraceae present in the diet, which contribute to the colonization of Rhodobacteraceae inside the *D. rerio* microbiome.

In summary, we revealed diet-specific alterations of gut microbial community compositions along with taxonomic co-occurrence networks and predicted metabolic profiles. Although we had minor limitations in the number of samples available, significant differences in microbial outcomes could be determined when we evaluated an SR diet using FPH as an established protein source in comparison with a diet using SCP in replacement of FPH in laboratory-reared *D. rerio.* Changes in the gut microbiome and associated predicted metabolic profiles are consistent with and support previously reported physiologic and transcriptomic outcomes [4]. Combined, these data may reveal potential health benefits of the consumption of bacterial-sourced protein, including comparable body weight gain, reduced fat deposition, and a higher percentage of fat-free lean tissue in fish fed the BP diet. The outcome of this study suggests a functional synergism between host and microbial metabolism and will lead to a greater understanding of the nutrient/health axis in *D. rerio.*<END ARTICLE>

### Author Contributions

The authors’ responsibilities were as follows – GBHG: formal data analysis and wrote the original draft of the manuscript; MBW: conducted animal experiments and wrote the original draft of the manuscript; JB: wrote the original draft of the manuscript; SBC conducted animal experiments and edited manuscript; CXF: data analytics and respective figures; CDM: high-throughput sequencing and reviewed and edited the manuscript; ALL: diet formulation and experimental design; AKB: data interpretation of the microbiome and wrote and edited the manuscript; SAW: overall supervision of the experiment design, data interpretation, supervised the animal experiments under the UAB IACUC animal use compliance, and edited manuscript; and all authors: read and approved the final manuscript.

### Conflict of interest

The authors report no knowledge of any conflicts of interest.

### Funding

This research was supported by the Microbiome Resource Center at the University of Alabama at Birmingham, School of Medicine: Comprehensive Cancer Center (P30AR050948), Center for Clinical Translational Science (UL1TR000165), the Heflin Center for Genomic Sciences, and the UAB Microbiome Center (to C.D.M.). This research was supported by Graduate Research Assistant funding from the Department of Biology, University of Alabama at Birmingham (to G.B.H.G.). This research was supported in part by the NIH NORC Nutrition Obesity Research Center (P30DK056336) (to S.A.W.) and Meridian Biotech, LLC.

### Data Availability

The high-throughput amplicon sequencing data sets of *D. rerio* samples are publicly available on the BioSample Submission Portal (https://www.ncbi.nlm.nih.gov/bioproject/) under the BioProject ID PRJNA958190.

### Ethics Statement

All animal experiments were conducted following the guidelines approved by the animal care and use under the Institutional Animal Care and Use Committee Permit IACUC-21893; 2021-Nov-2022 (S.A.W.), the University of Alabama at Birmingham.
